# Acute-Onset Bilateral External and Internal Ophthalmoplegia: A Rare Presentation of Miller Fisher Syndrome in a Pediatric Patient

**DOI:** 10.7759/cureus.75150

**Published:** 2024-12-05

**Authors:** Richa, Vineeta Pande, Amodini Arora, Md Owais Ali Khan, Shailaja Mane

**Affiliations:** 1 Pediatrics, Dr. D. Y. Patil Medical College, Hospital, and Research Centre, Dr. D. Y. Patil Vidyapeeth (Deemed to be University), Pune, IND; 2 Paediatrics, Dr. D. Y. Patil Medical College, Hospital, and Research Centre, Dr. D. Y. Patil Vidyapeeth (Deemed to be University), Pune, IND

**Keywords:** ataxia, diplopia, guillain-barré syndrome (gbs), miller fisher syndrome (mfs), ophthalmoplegia

## Abstract

Miller Fisher syndrome (MFS) is a rare Guillain-Barré syndrome (GBS) variant. The global incidence of GBS is approximately one to two in 100,000 children (aged 0 to 15 years) per year. Miller Fisher syndrome represents a further small subset, with the incidence being one to two in 1,000,000 children. It affects all age groups; however, adult males are more commonly affected, with a male-to-female ratio of 2:1. It usually presents with a triad of ataxia, areflexia, and ophthalmoplegia. The hallmark sign of MFS is ophthalmoplegia, with internal ophthalmoplegia being more common. Pupillary response may vary from sluggishly reactive to non-reactive. The external ophthalmoplegia observed in MFS is bilateral and symmetrical, but some unilateral cases have also been reported. Internal and external ophthalmoplegia occurring together in a pediatric patient has not been reported in the literature to the best of our knowledge. Thus, we are reporting this case to highlight the rare presentation of internal and external ophthalmoplegia in a pediatric patient.

Here, we present a case of a 10-year-old male child who presented with sudden onset ataxia, headache, blurring of vision, diplopia, and four-quadrant eye movement restriction. On examination, the child was overweight and had external and internal ophthalmoplegia (third, fourth, and sixth cranial nerve involvement) with ataxia and hypertension. There were no motor deficits or any other cranial nerve involvement. The GBS variant was considered the initial diagnosis. There was no history of previous infection. We investigated the case, and a lumbar puncture was done. Cerebrospinal fluid (CSF) analysis was normal, and anti-GQ1b antibodies were present. The patient was started on steroids and intravenous immunoglobulin (IVIG) and recovered slowly.

Most patients of MFS experience complete recovery within several weeks to months. Anti-GQ1b antibody positivity holds very crucial diagnostic value for MFS. IVIG and steroids are the treatments of choice for moderate to severe cases. This case report emphasizes the importance of suspecting and diagnosing MFS, particularly in pediatric patients and considering it as a differential diagnosis for acute-onset internal and external ophthalmoplegia with ataxia.

## Introduction

Guillain-Barré syndrome (GBS) comprises various types of acute immune-mediated polyneuropathies, with the most common being acute inflammatory demyelinating polyradiculoneuropathy (AIDP) and the rare variant being Miller Fisher syndrome (MFS). The global incidence of GBS is approximately one to two in 100,000 children (aged 0 to 15 years) per year. Miller Fisher syndrome represents a further small subset, with incidence being one to two in 1,000,000 [1]. Miller Fisher syndrome is estimated to be 1% to 7% of GBS cases in the West, but incidence is as high as 15% to 25% in Asia [2]. It affects all age groups; however, adult males are more commonly affected, with a male-to-female ratio of 2:1. It usually presents with a triad of ataxia, areflexia, and ophthalmoplegia [3]. The mechanism of MFS involves autoimmune-mediated peripheral neuropathy following exposure to a pathogen capable of molecular mimicry. The hallmark sign of MFS is ophthalmoplegia, with internal ophthalmoplegia being more common. Pupillary response may vary from sluggishly reactive to non-reactive [4]. Miller Fisher syndrome usually presents with bilateral and symmetrical external ophthalmoplegia. Rarely, unilateral cases have also been reported [5]. There can be isolated or combined involvement of the third, fourth, and sixth cranial nerves. The most common presenting symptom is diplopia, seen in 65% of patients [6]. The facial nerve can be involved in 30% of patients, resulting in weakness of orbicularis oculi and, consequently, lagophthalmos. Other common symptoms of MFS include gait abnormalities, limb weakness, and dysesthesias. Less common signs and symptoms include altered consciousness, ptosis, bulbar dysfunction, dysphagia, photophobia, dizziness, blurred vision, headache, facial weakness, and micturition abnormalities.

To the best of our knowledge, the literature does not document internal and external ophthalmoplegia occurring simultaneously in a pediatric patient. This lack of information could make it challenging to diagnose MFS in children. Thus, we are reporting this case to highlight the rare presentation of acute-onset internal and external ophthalmoplegia with ataxia with autonomic involvement (hypertension) in a pediatric patient.

## Case presentation

**Figure 1:**
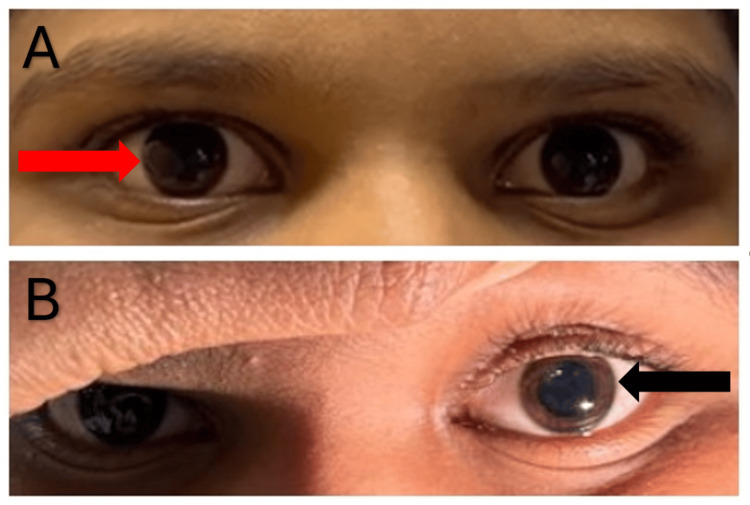
(A) The patient's eyes show bilateral mid-dilated and non-reactive pupils; (B) Improvement is seen on follow-up at seven weeks, where the iris is visible.

A 10-year-old immunized male child was brought to casualty with complaints of dizziness, diplopia, vomiting, and headache for two days. The patient was apparently well two days back when he developed dizziness, which was associated with difficulty in maintaining balance while walking, and frontal headache. There was no history of fever, loose stools, altered sensorium, photophobia, head trauma, recent drug intake, pain in the abdomen, bladder or bowel incontinence, back pain, or seizures. Past and birth history was uneventful. Developmentally, all milestones were achieved as per age. There was no history of similar complaints in the family. On anthropometric assessment, his weight was 55 kg, height was 142 cm, and body mass index (BMI) was 27.3 kg/m², indicating that he was overweight (gradually gained over four years). On first examination, the patient had tachycardia (heart rate (HR): 136/min) and hypertension (blood pressure (BP): 136/94 mmHg). Rest vital parameters were within normal limits. On central nervous system examination, higher mental functions were normal; there were bilateral, mid-dilated, fixed pupils not reacting to light (Figure 1) and bilateral eye movement restriction in all four directions, indicating third, fourth, and sixth nerve palsy.

**Figure 2:**
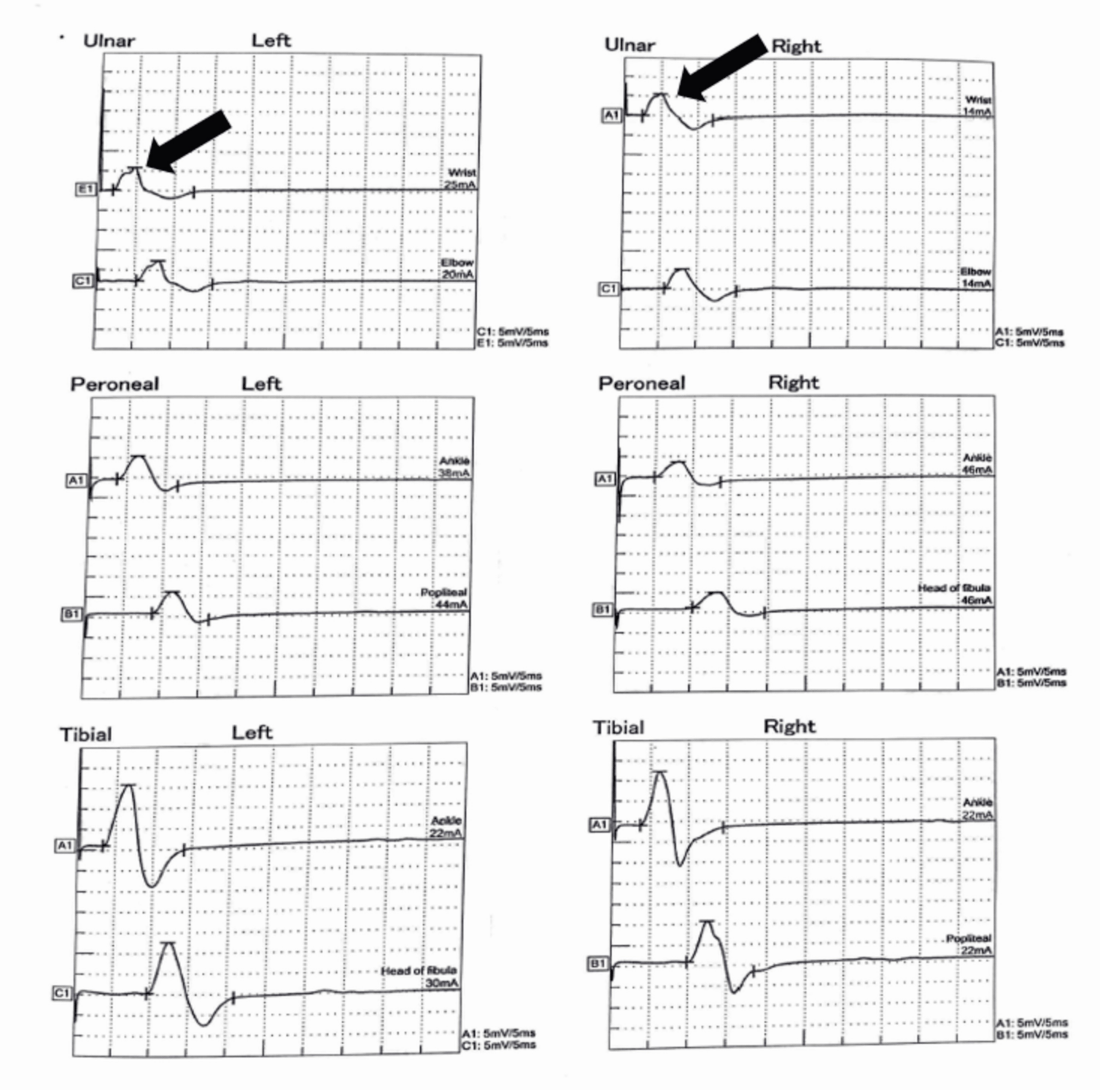
Reduced CMAP amplitude seen in the bilateral ulnar nerve on the motor nerve conduction study CMAP: compound muscle action potential

**Figure 3:**
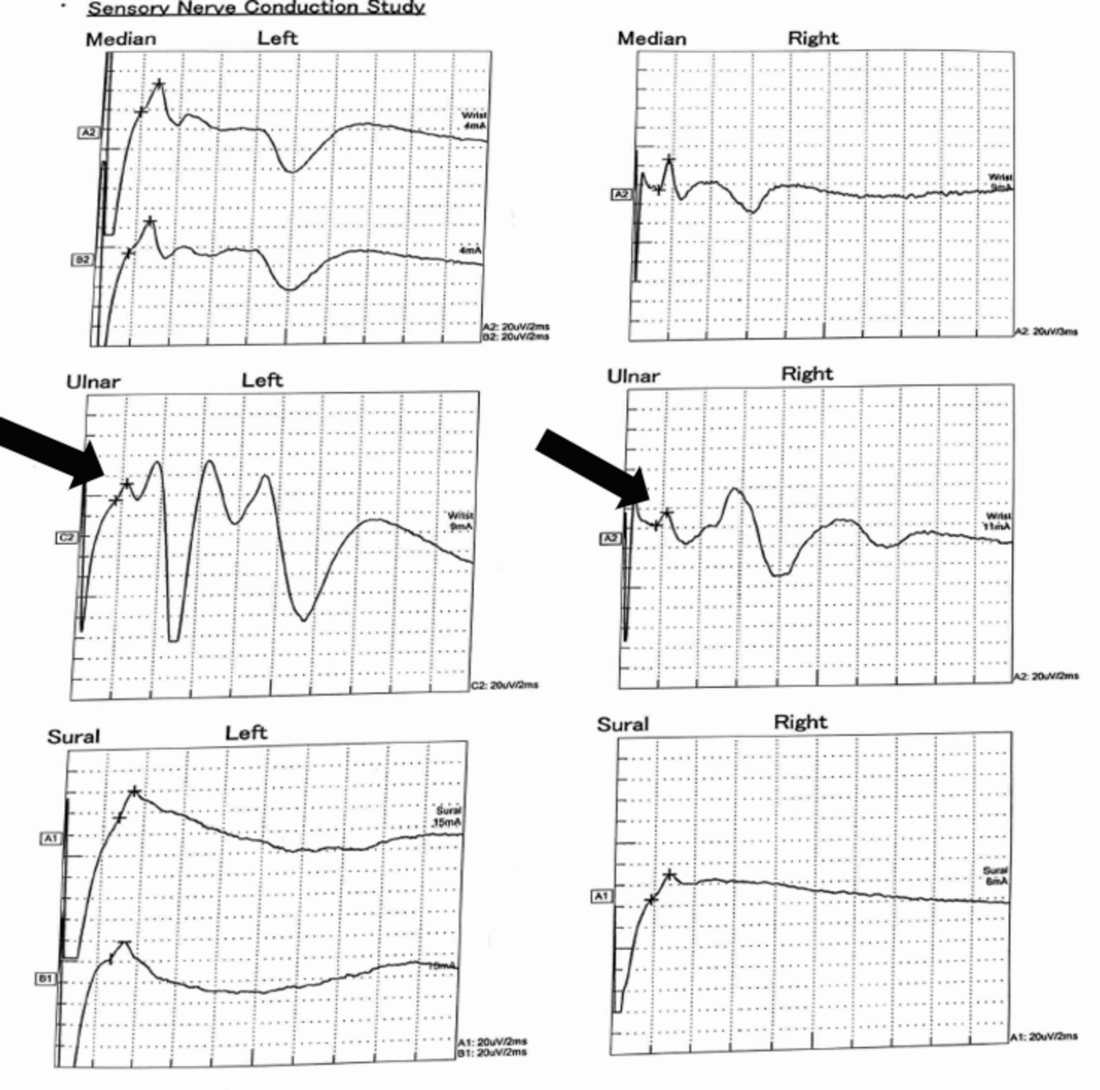
Reduced SNAP amplitude seen in the bilateral ulnar nerve on the sensory nerve conduction study SNAP: sensory nerve action potential

Other motor and sensory neurological examinations were within normal limits (tone, power, and deep tendon reflexes were normal in all limbs). Fundus examination showed no papilledema. A gag reflex was present, and cerebellar examination showed an inability to perform tandem walking. However, there was no dysdiadochokinesia, and the finger-nose test was normal. The GBS variant was considered as the initial differential diagnosis. The cerebrospinal fluid (CSF) opening pressure and routine microscopy were normal. Nerve conduction studies showed reduced compound muscle action potential (CMAP) and sensory nerve action potential (SNAP) amplitudes and impersistent F waves in bilateral ulnar nerves (Tables 1-2, Figures 2-3).

**Table 1:** Report of the nerve conduction studies performed on the patient: part one NCV: nerve conduction velocity

Site	Latency (ms)	Amplitude	Area	Stimulation (mA)	Segment	Distance (mm)	Interval (ms)	NCV (m/s)
Left median nerve					Temperature			
Wrist	2.0	28.7uV	1.4uVms	4.0	Wrist	110	2.0	55.0
	1.7	32.9uV	1.6uVms	4.0	-			
Right median nerve					Temperature			
Wrist	2.0	32.3uV	2.2uVms	9.0	Wrist	110	2.0	56.4
					-			
Left ulnar nerve					Temperature			
Wrist	1.7	16.4uV	0.5uVms	9.0	Wrist	100	1.7	57.5
Right ulnar nerve					Temperature			
Wrist	1.7	13.2uV	0.4uVms	11	Wrist	100	1.7	58.8
					Temperature			
Left sural nerve	2.8	25.1vV	1.9vVms	15	Sural		2.8	
					Temperature			
Right sural nerve	1.9	23.7uV	1.1uVm	6	Sural	80	1.9	43

**Table 2:** Report of the nerve conduction studies performed on the patient: part two NCV: nerve conduction velocity

Site	Latency (ms)	Duration (ms)	Amplitude	Area	Stimulation (mA)	Segment	Distance (mm)	Interval (ms)	NCV (m/s)
Left median nerve						Temperature			
Wrist	3.1	9.7	7.5mV	36.6mVms	23	*Wrist		3.1	
Elbow	6.2	9.6	7.2mV	33.7mVme	23.0	Wrist-elbow	190	3.2	60.3
						Elbow-axilla			
Right median nerve						Temperature			
Wrist	2.0	10.6	8.5mV	38.2mVms	23.0	*Wrist		2.0	
Ebow	6.5	10.5	7.0mV	33.0mVms	23.0	Wrist-elbow	190	3.6	52.8
						Elbow-axilla			
Left ulnar nerve						Temperature			
Wrist	20	10.7	5.6mV	19.7mVms	25.0	*Wrist		2.0	
Elbow	5.4	10.1	5.1mV	17.9mVms	20.0	Wrist-elbow	210	3.4	61.8
						Elbow-axilla			
Right ulnar nerve						Temperature			
Wrist	2.5	9.5	5.7mV	21.6mVms	14.0	*Wrist		2.5	
Elbow	5.7	9.7	4.9mV	20.8mVms	14.0	Wrist-elbow	210	3.2	65.6
						Elbow-axilla			
Left peroneal nerve						Temperature			
Ankle	3.7	8.0	5.7mV	21.0mVms	38.0	*Ankle		3.7	
Popliteal	8.8	7.4	5.2mV	17.6mVms	44.0	Ankle-popliteal	250	5.1	49.0
Right peroneal nerve						Temperature			
Ankle	5.0	8.8	3.8mV	15.3mVms	46.0	*Ankle		5.0	
Head of the fibula	10.3	9.3	3.7mV	16.4mVms	46.0	Ankle-head of the fibula	250	5.4	46.7
						Head of the fibula-popliteal			
Left tibial nerve						Temperature			
Ankle	3.4	10.7	14.8mV	67.5mVms	22.0	*Ankle		3.4	
Head of the fibula	9.7	11.4	12.3mV	57.7mVms	30.0	Ankle-head of the fibula	310	6.3	49.6
						Head of the fibula-popliteal			
Right tibial nerve						Temperature			
Ankle	3.7	10.9	13.2mV	52.9mVms	22.0	*Ankle		3.7	
Popliteal	9.9	8.6	9.9mV	38.5mVms	22.0	Ankle-popliteal	310	6.2	50.4

The hemogram and routine blood investigations were within normal limits. Low-density lipoprotein (LDL) was 140 mg/dl, and cholesterol was 200 mg/dl, which was borderline high. Renal Doppler was normal, and the antinuclear antibody (ANA) blot test was negative. Magnetic resonance imaging (MRI) of the brain and electroencephalogram (EEG) did not show any abnormality; 2D echocardiography showed mild left ventricular (LV) dysfunction with left ventricular ejection fraction (LVEF) of 45%. Urinary vanillylmandelic acid (VMA) levels were normal, and no abnormality was detected on ultrasonography of the abdomen.

In view of ophthalmoplegia with ataxia with nerve conduction studies report, a rare variant of GBS, MFS, was suspected (CSF anti-GQ1b antibodies were sent and reported positive), and the patient was started on intravenous immunoglobulin (IVIG) at 2 g/kg over five days along with intravenous dexamethasone. To control his hypertension, oral labetalol, enalapril, and amlodipine were started. At the one-week follow-up, there was mild improvement in ophthalmoplegia and ataxia. Additionally, his hypertension was under control. At the seven-week follow-up, there was a remarkable improvement in eye movements in all directions, with no ataxia and pupils being sluggishly reactive to light.

## Discussion

Around 30% of MFS patients may have atypical clinical manifestations. Anti-GQ1b antibodies are a crucial biomarker for the diagnosis of MFS. One distinctive aspect of MFS is the presence of anti-GQ1b antibodies in the majority of affected individuals [7]. These antibodies target the ganglioside GQ1b (abundant in the plasma membranes of the cranial nerves supplying the extraocular muscles and the presynaptic neuromuscular junctions) [5]. Testing for anti-GQ1b antibodies helps in narrowing down the differentials. Serological testing for the anti-GQ1b antibody is positive in 80%-95% of patients with MFS. According to research, anti-GQ1b testing is found to be superior to CSF studies in the first three weeks when suspecting MFS [8]. Other anti-glycoside antibodies, such as anti-GT1a, anti-LM1, anti-GD3, anti-GM1, anti-GM2, anti-GD1a, anti-GD1b, and anti-GalNAc, have also been implicated in the disease. The anti-GQ1b antibody syndromes include MFS, GBS, Bickerstaff brainstem encephalitis, and acute ophthalmoparesis without ataxia [9]. These differential diagnoses should be considered in patients with presentations that could be consistent with MFS, especially if the clinical picture is atypical or incomplete.

Neuroimaging is typically unremarkable in MFS. Rarely, nonspecific MRI abnormalities appear in 1% of patients [7]. The main purpose of neuroimaging is to exclude other potential etiologies like Bickerstaff brainstem encephalitis, brainstem stroke, and Wernicke's encephalopathy.

Cerebrospinal fluid studies in MFS reveal albuminocytological dissociation in a large majority of cases. However, it is important to note that albuminocytological dissociation in the CSF appears later in the disease course. Hence, the CSF analysis of our patient did not show this finding. Nerve conduction studies and electromyography (EMG) are often normal in MFS but can be useful in diagnosing other subtypes of GBS.

Miller Fisher syndrome is a self-resolving disease with an excellent prognosis [10]. The mean recovery time is about 10 weeks, but one-third of the patients may have residual neurological deficits [11]. Supportive care may be sufficient for mild cases. For moderate to severe cases, the treatment of choice for MFS is IVIG or plasmapheresis. Nearly all cases of ophthalmoplegia are resolved in six months [10]. The mortality rate is approximately 4%, with a 3% chance of recurrence [6]. Severe complications such as respiratory failure requiring mechanical ventilation are rare.

Medical therapies have yet to be proven effective, and IVIG does not have a significant impact on outcomes, though it does minimally hasten the onset of recovery from ophthalmoplegia [12]. Plasmapheresis has also shown some success in case reports, but retrospective studies do not validate this [13]. Corticosteroids alleviate neuropathic/radicular pain but have no impact on the recovery or outcome.

The diagnosis of MFS is primarily clinical after the exclusion of alternative etiologies and is confirmed later by additional laboratory testing [9]. Understanding the unique clinical presentation of MFS, especially in pediatric patients, is crucial for timely diagnosis and intervention. This case report elucidates the clinical presentation, diagnosis, and management of MFS, highlighting the significance of considering MFS in the differential diagnosis for acute-onset internal and external ophthalmoplegia with ataxia and autonomic involvement in a pediatric patient.

## Conclusions

Miller Fisher Syndrome is a rare variant of GBS. There’s a scarcity of available literature on MFS in the pediatric age group. Hence, it is not considered a usual differential in this age group. Our case thus highlights that MFS must always be suspected in cases with acute onset internal and external ophthalmoplegia in pediatrics. With early diagnosis and treatment, the progression of the disease can be halted, and focal neurological deficits can be prevented.
